# Transitions of dialysis status and outcomes after the unplanned first dialysis: a nationwide population-based cohort study

**DOI:** 10.1038/s41598-023-39913-w

**Published:** 2023-08-08

**Authors:** Chia-Te Liao, Jia-Hong Lai, Yu-Wei Chen, Yung-Ho Hsu, Mei-Yi Wu, Cai-Mei Zheng, Chih-Cheng Hsu, Mai-Szu Wu, Shao-Yuan Chuang

**Affiliations:** 1 Division of Nephrology, Department of Internal Medicine, Shuang Ho Hospital, Taipei Medical University, New Taipei City, Taiwan; 2 Division of Nephrology, Department of Internal Medicine, School of Medicine, College of Medicine, Taipei Medical University, Taipei, Taiwan; 3 TMU-Research Center of Urology and Kidney (TMU-RCUK), Taipei Medical University, Taipei, Taiwan; 4 Institute of Population Health Sciences, National Health Research Institutes, No. 35, Keyan Road, Zhunan, Miaoli County 35053 Taiwan; 5 Division of Nephrology, Department of Internal Medicine, Hsin Kuo Min Hospital, Taipei Medical University, Taoyuan City, Taiwan; 6 National Center for Geriatrics and Welfare Research, National Health Research Institutes, Yunlin, Taiwan

**Keywords:** Health care, Nephrology

## Abstract

In Taiwan, most first-time dialysis was started without the creation of an arteriovenous shunt. Here, we aimed to elucidate the transitions of dialysis status in the unplanned first dialysis patients and determine factors associated with their outcomes. A total of 50,315 unplanned first dialysis patients aged more than 18 years were identified from the National Health Insurance Dataset in Taiwan between 2001 and 2012. All patients were followed for 5 years for the transitions in dialysis status, including robust (dialysis-free), sporadic dialysis, continued dialysis, and death. Furthermore, factors associated with the development of continued dialysis and death were examined by the Cox proportional hazard models. After 5 years after the first dialysis occurrence, there were 5.39% with robust status, 1.67% with sporadic dialysis, 8.45% with continued dialysis, and 84.48% with death. Notably, we have identified common risk factors for developing maintenance dialysis and deaths, including male gender, older age, diabetes, coronary heart disease, stroke, heart failure, sepsis, and surgery. There was an extremely high mortality rate among the first unplanned dialysis patients in Taiwan. Less than 10% of these patients underwent continued dialysis during the 5-year follow-up period. This study highlighted the urgent need for interventions to improve patient outcomes.

## Introduction

Kidney disease is one of the major non-communicable diseases worldwide, leading to ever-increasing healthcare burden^1^. Kidney replacement therapy (KRT), including dialysis and transplantation, is an essential life-maintaining strategy to deal with functionally failed kidneys^2,3^. In the past, the outcome studies of the dialysis population have been focused on chronic kidney disease (CKD) stage 5D or end-stage kidney disease (ESKD) patients receiving maintenance dialysis^4,5^. More recently, observational studies emphasized the outcomes of the patients with acute kidney injury requiring dialysis (AKI-D)^6–9^. Notably, a high mortality rate within the first few months after dialysis initiation has been noticed in AKI-D patients, with the reported 28-day mortality rate around 30–70%^10–13^. Apart from death, the outcomes of those who have survived the AKI-D could be further categorized into three groups: ‘nonrecovery’ state with maintenance dialysis, ‘partial recovery’ state with a sporadic requirement of intermittent dialysis, and ‘recovery’ state without the necessity of dialysis^14,15^. The trajectories after AKI-D are diverse, which not only reflect the severity of AKI per se but also indicate the maladaptive tissue repair process underneath the kidney insults^16–18^. Hence, understanding the determinants of the fate after AKI-D could provide a basis for implementing preventive and therapeutic measures to halt or reverse the detrimental outcomes^19,20^.

Taiwan remains one of the countries with the highest incidence and prevalence rate of ESKD patients in the world^21^. It is estimated that the total expenditure for ESKD patients increased up to around 1.8 billion US dollars in 2017^22^. To reduce the dialysis burden through improving the quality of CKD care, the pre-end-stage renal disease pay for performance (pre-ESRD P4P) program has been implemented in Taiwan since 2006. Accumulating evidence has shown that the multidisciplinary care under this program has significantly improved patient survival and reduce the total cost for dialysis^23,24^. However, like other countries in the world, the comprehensive post-AKI care program in Taiwan has not been well addressed until very recently^25–27^. There is still a fundamental knowledge gap from the epidemiologic perspectives, with an insufficient study investigating the outcomes of patients with AKI-D at a nationwide scale in Taiwan.

Here, we conducted a retrospective observational study, utilizing National Health Research Dataset, to illustrate different outcome trajectories of patients receiving first dialysis in Taiwan, with a particular focus on the ‘unplanned dialysis’ group, which represented AKI-D patients. The transitions of their kidney outcomes in terms of dialysis requirement (i.e., a nonrecovery state with maintenance dialysis, partial recovery state with sporadic dialysis and dialysis-free recovery state) would be reported. Finally, we investigated the determinants for the development of continued dialysis and deaths after the first dialysis in this population.

## Methods

### Study population

In Taiwan, the National Health Insurance is a single-payer program that has operated since 1995, covering 98% of the population. The database includes patient demographics, diagnosis, and prescriptions in the hospital and in outpatient claims. Currently, the access and application of the National Health Insurance Research Database (NHIRD) was authorized by the Center for Welfare and Health Data Sciences (contact via the Center for Welfare and Health Data Sciences, https://dep.mohw.gov.tw/dos/np-2497-113.html) for researchers who meet the criteria for access to confidential data. The NHIRD is one of the largest nationwide population-based databases in the world. All insured persons’ medical records from 2000 to 2017 were included in the dataset for this study. For research purposes, the information for all persons was managed with a double scrambling protocol. The original identification number was encrypted to protect privacy while maintaining consistency. Therefore, it was possible to follow up patients by linking claims belonging to the same patient within the NHIRD datasets. This study was approved by the ethics committee of National Health Research Institute (NHRI, Institutional Review Board, EC1090308-E) and was carried out in accordance with the principles of the 2013 version of the Declaration of Helsinki 1975. The requirement that patients give informed consent was waived according to the regulation approved by the NHRI.

### Study design

We designed a cohort aged more than 18 years old and without dialysis from all members of the National Health Insurance Dataset. This cohort was followed from 1st January 2001 until 31st December 2017 to identify new dialysis for estimate the annual dialysis incidence, specific age groups and genders. The annual dialysis incidence was calculated with the numbers of adults with first dialysis divided by the numbers of adults in the middle of the year.

We further designed a 5-year-followed and fixed cohort which enrolled 158,880 unplanned first dialysis for describing the transitions of dialysis status between January 2001 and December 2012.

The recruited criteria of first dialysis patients occurred between 2001 and 2012 were as follows. Those who initiated their first dialysis after January 1st, 2013 (n = 153,997) were excluded, due to being unable to be followed for up to 5 years. Further exclusions included those with (1) peritoneal dialysis (n = 12,812), (2) renal transplant (n = 1955) during 2013 and 2017, and (3) death date error (n = 671).

We then classified all first dialysis patients (n = 208,662) into (1) planned dialysis (n = 23,785), (2) unplanned dialysis (n = 158,880) and unclassified group (n = 25,997). Those with either AV fistula (medical orders: 69032A, 69032B, 69032C) or AV grafts (medical orders: 69034C, 69038C) created before first dialysis without using permanent catheter (medical order: 69039B) or temporary (double lumen) catheter (medical order: 69006C) were classified into planned dialysis (n = 23,785). Unclassified dialysis was defined by those without AV fistula or AV grafts plus without permanent or temporary catheter. Those unclassified first dialysis patients (n = 25,997) had no recorders with procedure codes of catheter plus with AV fistula or AV grafts. The unplanned dialysis (n = 158,880) met with the presence of either permanent or double lumen catheter at the time of first dialysis (with or without AV shunt creation before first dialysis).

**Figure 1:**
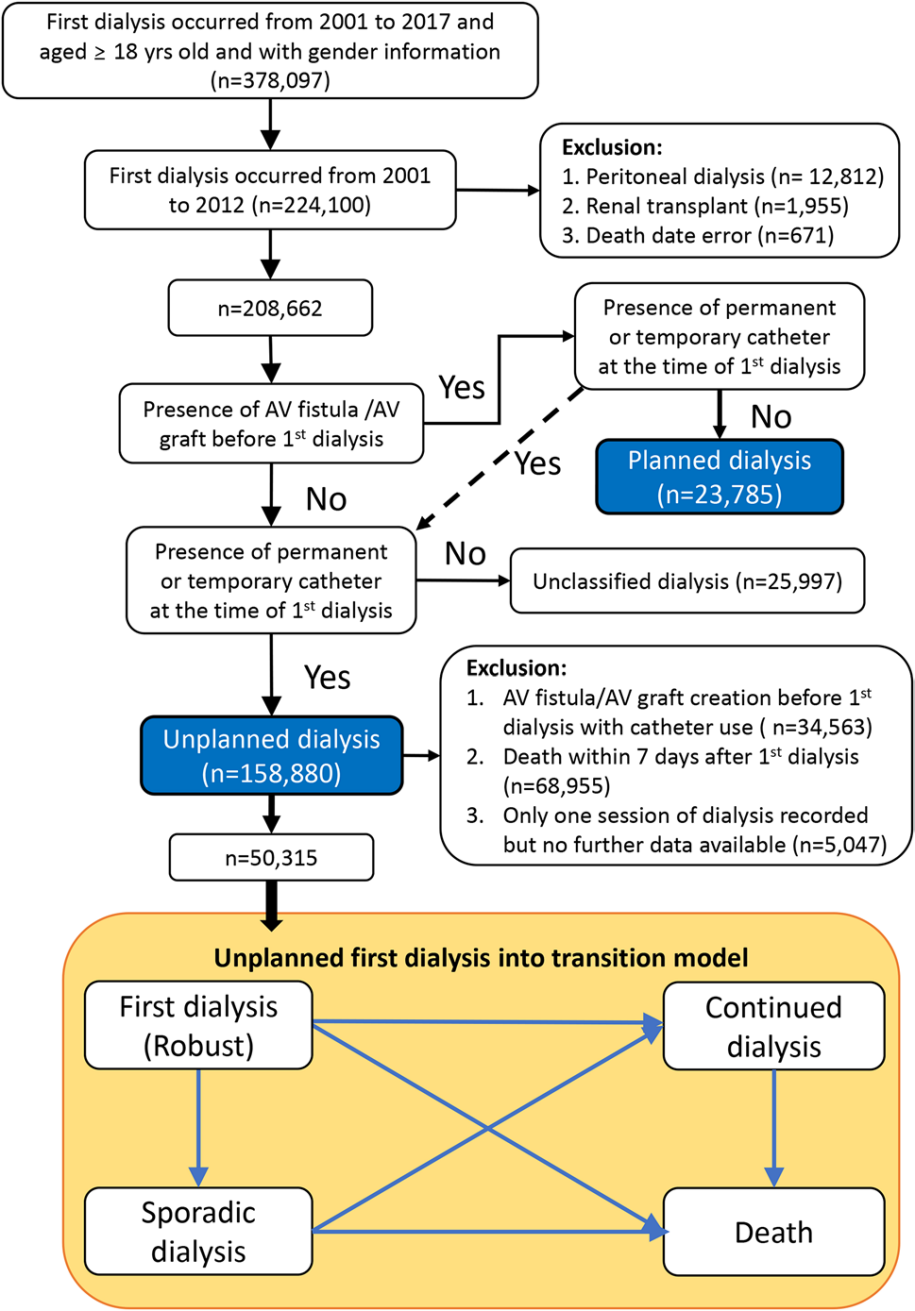
Flow chart of the study population and the protocol of selection strategy.

The transition model in the unplanned dialysis was described the transited stages of (1) temporary dialysis-free status (robust), (2) sporadic dialysis, (3) continued dialysis, and (4) final terminal death. Those patients met the exclusion criteria (a) arteriovenous shunt created before first dialysis but use of vascular catheter at the time of first dialysis (n = 34,563), (b) death in 7 days after first dialysis (n = 68,955), (c) free from dialysis after first dialysis (n = 5047), were not included for the transition cohort (n = 50,315) (Fig. 1).

### Definition of variables

All comorbidities were defined according to the diagnosis before the index date of the first dialysis by the medial recorders from the NHIRD in Taiwan.

Hypertension was defined as two outpatient claims with ICD-9-CM code 401–405. Dyslipidemia was defined as two outpatient claims with ICD-9-CM code 272. Diabetes was defined as two outpatient claims with ICD-9-CM code 250. Renal disease was defined as two outpatient visits with ICD-9-CM codes 580. Gout was defined as two outpatient visits with ICD-9-CM codes 274.9.

Cancer, stroke, coronary heart disease (CHD), and cardiovascular disease (CVD) also were identified from the hospital claim dataset. CHD was defined by ICD-9-CM codes 410–414. Stroke was defined by ICD-9CM codes 430–438. Heart failure (HF) was defined by ICD-9-CM codes 428. COPD was identified by 491.2. Sepsis was defined by ICD-9-CM codes with 038, 995, and 785.52. Surgery was defined by the medical recorders with surgical procedures. Death status was ascertained according to the National Death Registry system.

### Statistical methods

The continuous and categorical variables were presented by mean (± standard deviation) and proportions (numbers).

We used the Markov chain model to estimate the transit probabilities and the duration among stages. The Markov chain model has a feature that the predictive probability is only affected by the probability of current events and was not related to prior events. Therefore, the probability distribution at time *t* + 1 was only related to the events at time *t*, was no relation to the prior events and per sec values. The analysis of the Markov chain model was used the software *R* with the *msm* package (Fig. 1). In this transition model, we only recruited those patients with follow-up information about the transitions of dialysis stages or death. The determinants of transited to continued dialysis were explored and evaluated by the multivariate cox proportional hazard model. The independent determinants of death among those with unplanned dialysis were also identified by the cox multivariate regression model (Table 4) The survival analysis was conducted by the statistical software SAS 9.4.

## Results

### Trends of annual incidence of first dialysis in Taiwan

**Figure 2:**
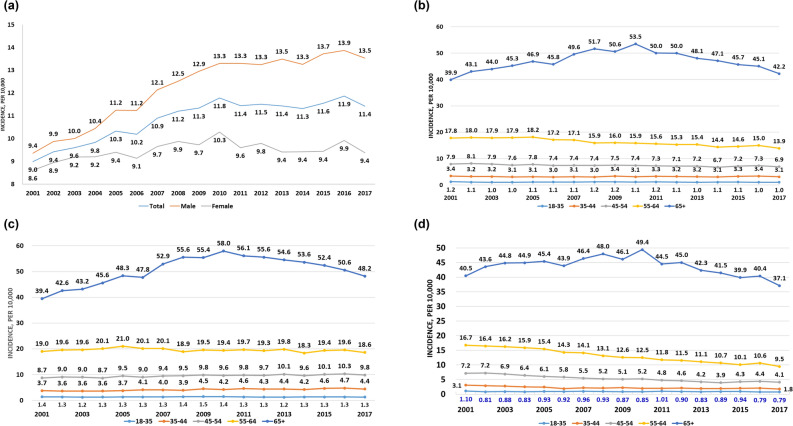
Trends of annual incidence of adult patients undergoing first dialysis in Taiwan, 2001–2017. (**a**) Stratified by sex, (**b**) stratified by age, (**c**) stratified by age in male adult patients, and (**d**) stratified by age in female adult patients.

The incidence risk of first dialysis continually increased in men from 2001 (9.36/10,000) to 2017 (13.53/10,000). However, the incidence of first dialysis increased from 2001 (8.61/10,000) until 2010 (9.38/10,000) in women and then the risk was relatively stable (Fig. 2a). The incidence rate of first dialysis among those aged > 65 years increased from 2001 until 2010, then a decline trend until 2017, whilst the declining trend presented among those aged less than 65 years (Fig. 2b). Among men, the increased trends were found among those aged between 35 and 44 years and between 45 and 54 years (Fig. 2c). Among women, the decreasing trends were found among women aged less than 65 years (Fig. 2d). Both men and women with aged > 65 years had an increased trend of first dialysis incidence before 2010, then a continually decline trend thereafter (Fig. 2c,d).

### Clinical characteristics of the planned and unplanned first dialysis

Within 397,601.18 person-years [PYs], 9985 planned dialysis patients and 115,994 unplanned dialysis patients died respectively. The unplanned dialysis patients had a higher mortality rate (377.67 vs. 110.37 per 1000 PYs, p < 0.0001) and 5-year death risk (73.01% vs. 41.98%, p < 0.0001) than the planned dialysis patients (Supplementary Table 1). Those patients with the planned dialysis had higher proportions of comorbidities, including hypertension, diabetes, dyslipidemia, but low prevalence rates of stroke, CHD, heart failure, COPD, sepsis, and surgery, compared to those with the unplanned dialysis (Supplementary Table 1). Among unplanned dialysis, the average age was 66.78% and the prevalence rates of renal disease, hypertension, diabetes, coronary heart disease, stroke and heart failure were 57.81%, 83.52%, 60.66%, 53.29%, 41.52% and 39.77%, respectively.

Among the unplanned dialysis patients, 67,024 had no prior history of kidney disease, and 91,856 had pre-existing kidney disease. Those without kidney history had a higher mortality rate (598.28 vs. 285.86 per 1000 PYs, p < 0.0001) and 5-year death risk (80.56% vs. 67.50%, p < 0.0001) than those with kidney disease (Supplementary Table 2). Those without prior kidney disease had a lower prevalence in hypertension, diabetes, dyslipidemia, CHD, stroke, heart failure, COPD, and gout than those with pre-existing kidney disease (Supplementary Table 2).

### Transitions of the unplanned first dialysis

We further conducted a transition model to describe the transit pathways among the unplanned dialysis patients (n = 50,315). The mean age was 68.6 years old. The prevalence of comorbidities, including renal disease, hypertension, diabetes, dyslipidemia, CHD, stroke, heart failure, COPD, gout, sepsis, and surgery were presented in Table 1. Four stages after first dialysis included stage 0—robust (without further dialysis), stage 1—sporadic dialysis, stage 2—continued dialysis, and stage 3—death. Stage 2 (continued dialysis) had the longest sojourn time with an average of 4.21 years for men and 5.89 years for women. Stage 1 (sporadic dialysis) had the shortest sojourn time among all groups.

**Table 1:** Clinical characteristics of the unplanned first dialysis patients entered the transition model (n = 50,315).

Variables	Proportion (n)/mean ± standard deviation
Age, years	68.58 ± 13.34
Male gender, %	47.29% (23,794)
Kidney disease, %	65.45% (32,931)
Hypertension, %	88.28% (44,418)
Diabetes, %	67.26% (33,842)
Dyslipidemia, %	52.78% (26,556)
CHD, %	58.08% (29,223)
Stroke, %	45.82% (23,054)
Heart failure, %	44.46% (22,370)
COPD, %	13.49% (6787)
Gout, %	27.32% (13,746)
Sepsis, %	32.49% (16,347)
Surgery, %	85.30% (42,919)

*CHD* coronary heart disease, *COPD* chronic obstructive pulmonary disease.

After 6 months of first dialysis occurrence, there was 74.67% for robust, 3.10% for sporadic, 3.70% into the continued dialysis, and 18.52% died. After 1 year, there was 55.76% for robust, 4.48% for sporadic, 6.34% into the continued dialysis, and 33.42% died. After 3 years, only 17.34% of patients were robust and 3.66% for sporadic, 9.90% into the continued dialysis, and 69.10% died. However, after 5 years of first dialysis occurrence, only 5.39% remained to stay free from dialysis, 1.67% for sporadic stage, 8.45% was into continued dialysis, and 84.48% died (Table 2).

**Table 2:** Transition rates of multiple stages of dialysis status among the unplanned first dialysis patients entered the transition model (n = 50,315).

	Stage 0 (no dialysis) (%)	Stage 1 (sporadic) (%)	Stage 2 (continued) (%)	Stage 3 (death) (%)
After 6 months	74.67	3.10	3.70	18.52
After 1 year	55.76	4.48	6.34	33.42
After 3 years	17.34	3.66	9.90	69.10
After 5 years	5.39	1.67	8.45	84.48
Male				
After 6 months	74.43	3.15	3.65	18.77
After 1 year	55.39	4.55	6.23	33.82
After 3 years	17.00	3.71	9.70	69.60
After 5 years	5.22	1.69	8.24	84.86
Female				
After 6 months	82.82	2.00	4.53	10.65
After 1 year	68.59	3.04	8.19	20.17
After 3 years	32.27	3.12	15.82	48.79
After 5 years	15.19	1.85	16.47	66.50
Age: 18–45				
After 6 months	74.45	3.16	3.61	18.78
After 1 year	55.43	4.56	6.18	33.83
After 3 years	17.03	3.72	9.61	69.64
After 5 years	5.23	1.70	8.15	84.92
Age: 45–64				
After 6 months	79.24	2.45	4.37	13.94
After 1 year	62.78	3.63	7.71	25.88
After 3 years	24.75	3.32	13.49	58.44
After 5 years	9.76	1.73	12.76	75.75
Age: 65 +				
After 6 months	71.24	3.57	3.30	21.89
After 1 year	50.75	5.04	5.51	38.70
After 3 years	13.07	3.74	7.91	75.28
After 5 years	3.37	1.54	6.25	88.84

The transition rates of multiple stages of dialysis status stratified by genders and age groups presented in Table 2. Male patients had higher risk for death than women (18.77% vs. 10.65%) after 6 months and during all periods of follow-up. On the contrary, higher proportion of women entered continued dialysis than men during all periods of follow-up.

### Determinants for the development of continued dialysis and death after the first unplanned dialysis

We conducted a Cox proportional model to identify the determinants of developed to continued dialysis after the first unplanned dialysis and follow-up for 5 years or until the end of the study. The median time of follow up was 1.27 years and the total follow time was 732,458.34 PYs. Male gender, older age, diabetes, kidney disease, CHD, stroke, HF, sepsis and surgery were positively associated with the development of continued dialysis, and hypertension, dyslipidemia, gout, and COPD were negatively associated with the development of continued dialysis (Table 3).

**Table 3:** The determinants of developed into continued dialysis after the first dialysis among the unplanned dialysis patients entered the transition model (n = 50,315).

	Model-1			Model-2		
	Hazard rate	95% CI	p-value	Hazard rate	95% CI	p-value
Male vs. Female	1.207	(1.201, 1.214)	< 0.0001	1.207	(1.201, 1.213)	< 0.0001
Age						
45–64 vs. 18–44, years	1.401	(1.384, 1.418)	< 0.0001	1.580	(1.563, 1.598)	< 0.0001
65 + vs. 18–44, years	2.280	(2.253, 2.308)	< 0.0001	2.489	(2.462, 2.516)	< 0.0001
Hypertension	0.582	(0.579, 0.585)	< 0.0001	0.650	(0.647, 0.654)	< 0.0001
Dyslipidemia	0.670	(0.665, 0.676)	< 0.0001	0.742	(0.736, 0.748)	< 0.0001
Diabetes	1.062	(1.056, 1.069)	< 0.0001	1.148	(1.142, 1.155)	< 0.0001
Gout	0.910	(0.905, 0.916)	< 0.0001	0.947	(0.942, 0.952)	< 0.0001
Kidney disease	1.081	(1.075, 1.088)	< 0.0001	1.078	(1.072, 1.084)	< 0.0001
CHD	1.143	(1.137, 1.15)	< 0.0001	1.121	(1.115, 1.127)	< 0.0001
Heart failure	1.113	(1.107, 1.12)	< 0.0001	1.104	(1.099, 1.11)	< 0.0001
Stroke	1.101	(1.093, 1.11)	< 0.0001	1.080	(1.073, 1.088)	< 0.0001
COPD	0.991	(0.985, 0.997)	0.0182	0.988	(0.983, 0.994)	0.0024
Sepsis	1.660	(1.651, 1.669)	< 0.0001	1.531	(1.523, 1.539)	< 0.0001
Surgery	1.626	(1.612, 1.64)	< 0.0001	1.433	(1.423, 1.443)	< 0.0001

*CHD* coronary heart disease, *COPD* chronic obstructive pulmonary disease.

Model-1: All patients were followed 5 years.

Model-2: All patients were followed until the end of the study. The median time of follow up was 1.27 years, and the total follow person-years was 732,458.34.

Similarly, we performed another Cox proportional model to determine factors associated with the deaths after the first unplanned dialysis. Notably, we found that Male gender, older age, diabetes, CHD, stroke, HF, COPD, sepsis, and surgery were positively associated with the mortality after first dialysis, whilst kidney disease, hypertension, and dyslipidemia were negatively associated with the mortality after first dialysis (Table 4).

**Table 4:** The determinants of the deaths after the first unplanned dialysis (n = 158,880).

	Hazard ratio	Low of 95% CI	Up of 95% CI	p-value
Male vs. female	1.178	1.164	1.192	< 0.0001
Age				
45–64 vs. 18–44, years	1.416	1.378	1.455	< 0.0001
65 + vs. 18–44, years	2.226	2.168	2.286	< 0.0001
Kidney disease	0.631	0.624	0.639	< 0.0001
Hypertension	0.679	0.667	0.691	< 0.0001
Diabetes	1.025	1.011	1.038	0.0003
Dyslipidemia	0.921	0.909	0.933	< 0.0001
CHD	1.068	1.054	1.083	< 0.0001
Stroke	1.108	1.094	1.122	< 0.0001
Heart failure	1.077	1.063	1.09	< 0.0001
COPD	1.08	1.062	1.098	< 0.0001
Gout	1.001	0.987	1.015	0.8842
Sepsis	1.609	1.59	1.629	< 0.0001
Surgery	1.268	1.239	1.298	< 0.0001

*CHD* coronary heart disease, *COPD* chronic obstructive pulmonary disease.

Finally, we noticed that those patients who remained free from dialysis after first unplanned dialysis were younger, female predominance, fewer comorbid illness, and particularly very low proportion of surgery (Supplementary Table 3).

## Discussion

In this study, we illustrated the increasing trend of the annual incidence of adult patients receiving first dialysis treatment in Taiwan between 2001 and 2017 and demonstrated gender- and age-specific differences with the trends, respectively. Further comparisons between patients with the ‘planned dialysis’ and the ‘unplanned dialysis’ have revealed that the latter has a higher death risk and mortality rate. Notably, the death risk and mortality rate are higher in the ‘unplanned dialysis’ group without previous kidney disease than those with previous kidney disease. Additionally, we have characterized the transition pathways of the ‘unplanned first dialysis’, which has disclosed that only 5.39% retained a dialysis-free status by the end of the 5-year follow-up period. Finally, we have identified common risk factors for developing maintenance dialysis and death, including male gender, older age, diabetics, CHD, stroke, HF, and sepsis.

Our data has shown that patients aged more than 65 years experienced the highest incidence rate of receiving first dialysis treatment. Interestingly, there is a slight downturn after 2010 in the elderly of both genders. One possible explanation is that the amount of acute dialysis initiation in critically ill patients with grave prognoses has been reduced. Thus the ‘futility dialysis’ was limited^28^. Of note, there is still an increasing incidence rate of ESKD during the same period, especially among men aged more than 65 years and women aged more than 75 years^22^. Taken together, it is suggested that the proportion of the elderly receiving maintenance dialysis therapy is continuedly increasing, which could be attributable to the increasing aged population. In addition, the implementation of pre-ESRD care would delay the timing of the first dialysis treatment and prolong their lifespan^24^.

When we stratified the first dialysis cohort into the ‘planned’ (AV shunt creation before the first dialysis) and the ‘unplanned’ dialysis, we found that they have distinctive clinical characteristics at baseline. Briefly, those with the ‘planned dialysis’ had more chronic medical conditions such as hypertension, diabetics, dyslipidemia, while those with the ‘unplanned dialysis’ more likely had acute medical illnesses such as stroke, CHD, HF, and sepsis. Hence, the ‘unplanned dialysis’ group could be considered as AKI-D, which primarily came from pre-existing CKD patients with acute renal dysfunction (Acute on CKD) and new-onset severe AKI patients without previous kidney disease (de novo AKI). Our results revealed that those with ‘unplanned dialysis’ but no history of kidney disease had a significantly higher mortality rate than those with a history of kidney disease. Notably, the former had more surgical procedures and sepsis before their first dialysis compared to the latter, suggesting that these two factors are the key determinants of the survival chances within the de novo AKI-D.

In contrast to the ‘planned dialysis’ with either entering continued dialysis or deaths onwards, those with the ‘unplanned dialysis’ could be separated into four transition stages (survived without dialysis, sporadic dialysis, continued dialysis, and death) during the follow-up period. Our results showed that there is a high mortality rate (43.4%, n = 68,955, Fig. 1) within the 7-day period after the first dialysis in patients with the ‘unplanned dialysis’. This finding is consistent with the previously reported high mortality rate in AKI-D patients worldwide, suggesting more considerations should be taken while dealing with acute critically ill patients, especially the elderly with multiple comorbid conditions, where a conservative approach should be encouraged to avoid the futility dialysis^29^. Notably, after surviving the first 7-day period, around three-fourths of those with the ‘unplanned dialysis’ survived without further dialysis at 6-month follow-up, but the proportion declined to 55% at 1 year, about 17% at 3 years, and around 5% at 5 years. The transitions from dialysis-free status to continued dialysis and to death were obvious, with more than 80% death by the end of the 5-year period. Hence, there is an urgent need for strengthening the post-AKI care in Taiwan, to improve the AKI outcomes^25^. However, due to the complexity of the post-AKI scenario, it is believed that more evidence-based risk prediction, stratification, and validation will be needed to achieve high-quality post-AKI care in the future^30^. Finally, we have identified the potential risk factors for developing continued dialysis and death in patients with ‘unplanned dialysis’. It is shown that male gender, older age, diabetes, CHD, stroke, HF, and sepsis are strong risk factors for both continued dialysis and death. On the contrary, patients who recovered and remained dialysis-free after the first dialysis were more likely younger, female, and less likely to have pre-existing kidney disease, other major comorbidities, and sepsis.

The main strength of this study is using nationwide population-based data to longitudinally follow up the patients receiving the first dialysis in Taiwan. However, this study was constrained by the retrospective nature of the design. Therefore, our observations might have been affected by confounding factors and not be generalizable to patients elsewhere. Besides, the lack of relevant biochemistry tests such as serum creatinine has hampered the rigorous definition of kidney disease status (de novo AKI, AKI on CKD, late-stage CKD) at the time of receiving the first dialysis. Additionally, utilization of “procedure-based” approach to stratify the planned dialysis and unplanned dialysis groups would have overlapping issues. For example, those patients who had AV shunt creation before first dialysis but used vascular catheters for their first dialysis had difficulty justifying the unexpected/unplanned nature due to lack of detailed clinical information. Finally, lack of detailed medications, which might affect patients’ outcome as well.

In conclusion, this is the first time a study has addressed the incidence and transitional outcomes of all patients initiating the first dialysis at a nationwide scale in Taiwan. In agreement with other studies, those with AKI-D had a higher mortality rate than those with late-stage CKD (the ‘planned dialysis’). Up to nearly 95% of all patients who survived after first dialysis without further dialysis eventually developed to continued dialysis and death at the end of 5-year follow-up, indicating more efforts on post-AKI care at a national scale is warranted to improve the renal and overall survivals.

### Supplementary Information

Supplementary Tables.

## Data Availability

Data from the National Health Insurance Dataset in Taiwan and from the National Death Registry cannot be shared publicly because of the Law of Personal Information Protection in Taiwan. Data are available from the Center for Welfare and Health Data Sciences (contact via the Center for Welfare and Health Data Sciences, https://dep.mohw.gov.tw/dos/np-2497-113.html) for researchers who meet the criteria for access to confidential data.
