# Supplementary material for: Transitions of dialysis status and outcomes after the unplanned first dialysis: a nationwide population-based cohort study
Source: Sci Rep. 2023 Aug 8;13:12867. doi: 10.1038/s41598-023-39913-w (PMC10409749; doi:10.1038/s41598-023-39913-w)
Supplement: Supplementary file 1 — Supplementary Tables. [file 41598_2023_39913_MOESM1_ESM.docx]

## Title: Transitions of dialysis status and outcomes after the unplanned first dialysis:

## A nationwide population-based cohort study

Chia-Te Liao^1,2,3^, Jia-Hong Lai^4^, Yu-Wei Chen^1,2,3^, Yung-Ho Hsu^1,2,3,5^, Mei-Yi Wu^1,2,3^, Cai-Mei Zheng^1,2,3^,

Chih-Cheng Hsu^4^, Mai-Szu Wu^1,2,3*^, Shao-Yuan Chuang^4*^

**Supplemental tables**

**Supplemental table 1.** Clinical characteristics of the planned and unplanned dialysis patients in the first dialysis cohort

|  | Planned dialysis (n=23,785) | | Unplanned dialysis (n=158,880) | | p-value |
| --- | --- | --- | --- | --- | --- |
| Age, yrs | 63.44+-12.30 | | 66.87+-14.60 | | <0.0001 |
| Male gender, % | 53.27% | (12,671) | 56.52% | (89,793) | <0.0001 |
| Renal disease, % | 98.60% | (23,452) | 57.81% | (91,856) | <0.0001 |
| Hypertension, % | 95.38% | (22,687) | 83.52% | (132,693) | <0.0001 |
| Diabetes, % | 68.40% | (16,269) | 60.66% | (96,382) | <0.0001 |
| Dyslipidemia, % | 60.86% | (14,475) | 48.00% | (76,268) | <0.0001 |
| CHD, % | 51.55% | (12,260) | 53.29% | (84,668) | <0.0001 |
| Stroke, % | 33.59% | (7,989) | 41.52% | (65,968) | <0.0001 |
| Heart failure, % | 36.19% | (8,608) | 39.77% | (63,184) | <0.0001 |
| COPD, % | 7.24% | (1,723) | 13.63% | (21,655) | <0.0001 |
| Gout/hyperuricemia, % | 29.42% | (6,998) | 25.22% | (40,063) | <0.0001 |
| Sepsis, % | 13.10% | (3,115) | 40.15% | (63,795) | <0.0001 |
| Surgery, % | 45.58% | (10,841) | 92.03% | (146,215) | <0.0001 |
|  |  | |  | |  |
| Follow-up periods, yrs | 90,466.71 | | 307,134.47 | |  |
| Events of death, | 9,985 | | 115,994 | |  |
| Mortality, per 1000 PYs | 110.37 | | 377.67 | |  |
| 5-year mortality, % | 41.98% | | 73.01% | |  |

**Supplemental table 2.** Clinical characteristics of the study population in the unplanned first dialysis with and without kidney diseases (n=158,880)

|  | Without kidney disease (n=67,024) | | With kidney disease (n=91,856) | | p-value |
| --- | --- | --- | --- | --- | --- |
| Age, yrs | 65.66+-15.86 | | 67.75+-13.54 | | <0.0001 |
| Male gender, % | 61.6% | (41,286) | 52.81% | (48,507) | <0.0001 |
| Renal disease, % |  |  |  |  |  |
| Hypertension, % | 73.3% | (49,150) | 90.95% | (83,543) | <0.0001 |
| Diabetes, % | 52.6% | (35,253) | 66.55% | (61,129) | <0.0001 |
| Dyslipidemia, % | 40.82% | (27,356) | 53.25% | (48,912) | <0.0001 |
| CHD, % | 47.89% | (32,098) | 57.23% | (52,570) | <0.0001 |
| Stroke, % | 39.09% | (26,201) | 43.29% | (39,767) | <0.0001 |
| Heart failure, % | 32.38% | (21,700) | 45.16% | (41,484) | <0.0001 |
| COPD, % | 14.01% | (9,387) | 13.36% | (12,268) | 0.0002 |
| Gout, % | 19.74% | (13,229) | 29.21% | (26,834) | <0.0001 |
| Sepsis, % | 49.47% | (33,158) | 33.35% | (30,637) | <0.0001 |
| Surgery, % | 93.99% | (62,995) | 90.6% | (83,220) | <0.0001 |
|  |  | |  | |  |
| Follow-up periods, yrs | 90,250.23 | | 216,884.24 | |  |
| Events of death, | 53,995 | | 61,999 | |  |
| Mortality, per 1000 PYs | 598.28 | | 285.86 | |  |
| 5-year mortality, % | 80.56% | | 67.50% | |  |

**Supplemental table 3.** Comparison of clinical characteristics among those who were free from dialysis, early mortality (died within 7 days) and those entered transition model after first dialysis

| Variables | Free from dialysis  (N=5,047) | | Died within 7 days  (N=68,955) | | Transition model  (N=50,315) | |
| --- | --- | --- | --- | --- | --- | --- |
| Age, yrs | 53.63±16.86 | | 68.29±15.10 | | 68.58±13.34 | |
| Male gender, % | 36.6% | (1847) | 39.47% | (27219) | 47.29% | (23794) |
| Kidney disease, % | 17.38% | (877) | 40.63% | (28016) | 65.45% | (32931) |
| Hypertension, % | 56.81% | (2867) | 77.65% | (53547) | 88.28% | (44418) |
| Diabetes, % | 39.45% | (1991) | 53.90% | (37168) | 67.26% | (33842) |
| Dyslipidemia, % | 39.47% | (1992) | 42.00% | (28958) | 52.78% | (26556) |
| CHD, % | 32.45% | (1638) | 52.33% | (36086) | 58.08% | (29223) |
| Stroke, % | 23.18% | (1170) | 42.86% | (29551) | 45.82% | (23054) |
| Heart failure, % | 16.23% | (819) | 38.17% | (26320) | 44.46% | (22370) |
| COPD, % | 5.92% | (299) | 16.85% | (11621) | 13.49% | (6787) |
| Gout, % | 20.65% | (1042) | 24.05% | (16582) | 27.32% | (13746) |
| Sepsis, % | 36.3% | (1832) | 55.77% | (38456) | 32.49% | (16347) |
| Surgery, % | 2.03% | (4645) | 93.87% | (64725) | 85.30% | (42919) |
